# Adherence to oral HIV pre-exposure prophylaxis among female sex workers in Kampala, Uganda

**DOI:** 10.4314/ahs.v21i3.12

**Published:** 2021-09

**Authors:** Grace Kakoola Nalukwago, John Bosco Isunju, Timothy Muwonge, Thomas Katairo, Nelson Bunani, Fred Semitala, Peter Kyambadde, Flavia Matovu

**Affiliations:** 1 Makerere University College of Health Sciences, Public Health; 2 Makerere University Infectious Diseases Institute, Research; 3 Makerere University College of Health Sciences, School of Medicine, Clinical Epidemiology Unit; 4 Most at Risk Population Iniatives (MarPI); 5 Makerere University School of Public Health, Epidemiology and Biostatistics; 6 MU-JHU Research Collaboration

**Keywords:** Oral PrEP, adherence, female sex workers

## Abstract

**Introduction:**

In Kampala Uganda, female sex workers (FSWs) have high HIV prevalence (33%). Oral PrEP is a novel HIV prevention intervention that offers hope to decrease HIV incidence in key populations especially among FSWs. Studies have shown that with poor adherence, oral PrEP has no efficacy, and therefore adherence to PrEP is critical among FSWs to maximize HIV prevention. However, implementation data on adherence to PrEP among FSWs is limited so this study sought to assess adherence to PrEP. Specifically, we sought to 1) determine the level of adherence to PrEP among FSWs, and 2) determine factors associated with PrEP adherence.

**Methods:**

This cross-sectional study was conducted from November to December 2018; 126 FSWs using PrEP were interviewed using a questionnaire. Adherence was categorically defined as high adherence and low adherence. Logistic regression was done.

**Results:**

Using long-term contraception methods (OR 0.06, 95% CI: 0.04–0.77) and not using condoms with clients (OR 0.07, 95% CI: 0.01–0.42) were negatively associated with high PrEP adherence.

**Conclusion:**

Barriers to PrEP adherence need to be addressed for successful PrEP implementation to improve adherence going forward. Service care providers should reinforce positive behaviors such as use of condoms devotedly during PrEP breaks.

## Introduction

Sub-Saharan Africa remains severely affected by Human Immune Deficiency syndrome (HIV), with nearly 1 in every 25 adults (4.2%) living with HIV^1^. In Uganda, approximately 1.2 million people were living with HIV in 2016 2.

The HIV epidemic in Uganda among people aged 15 to 64 years continues to disproportionately affect women with a prevalence of 7.5% compared to 4.3% among males. This is more so among women in urban areas as compared to the rural areas, with a prevalence of 9.8% and 6.7% respectively^2^. Nearly one fifth (11.5% to 18.6%) of HIV infections in women are attributed to sex work^3^, ^4^, with the HIV prevalence among female sex worker (FSW)s in Kampala as high as 33%^5^. Given this disproportionate burden of HIV, innovative HIV prevention interventions are particularly important to lower the risk of HIV for FSWs.

Oral pre-exposure prophylaxis (PrEP) is a novel biomedical HIV prevention intervention recommended for use by World Health Organization (WHO) among high risk populations including FSWs^6^. Oral tenofovir disoproxil fumarate (TDF) is the currently recommended PrEP regimen for women; this is an anti-retroviral medication which is recommended to be taken daily. It offers a locus of control for women to be in charge of their HIV prevention choices.

In the Partners Pre-Exposure Prophylaxis study, there was a highly significant protective effect of PrEP among female participants^7^. Among uninfected controls, drug-level analyses revealed adherence rates of 81%, and 83% in the TDF-FTC and in the TDF group respectively^7^. Indeed PrEP efficacy is highly dependent on good adherence to the medication^7^,^8^; with poor adherence, oral PrEP has no efficacy^9^. PrEP adherence has been studied in the context of clinical trials, for other at-risk populations such as sero-discordant couples and among men who have sex with men (MSM), but little is known about factors associated with adherence in real world settings, particularly for FSWs.

In Uganda PrEP roll-out started in August 2017 in a phased way, initially offered at public health facilities that provide care to high risk populations. PrEP is being offered to persons at substantial risk who are referred to as key populations (KP) and priority populations; these include FSWs, men who have sex with men (MSM), fisher-folk, and sero-discordant couples^10^. Since PrEP roll out started in Uganda, FSWs have been the majority of PrEP users in the country^11^.

Clinical trial studies conducted elsewhere have reported factors that facilitate PrEP use and adherence among high-risk populations to include certain drug characteristics, and the ability to use PrEP secretly or when condom use may be challenging, as well as perceived support from study staff, family and friends. Relatedly studies further report drug-related issues, such as side-effects and pill characteristics; logistical issues around drug use, such as timely refills and travel; and social stigma as barriers to PrEP use among potential PrEP users^12^,^13^. These studies have been conducted in different settings, and such factors have not been studied in Uganda among people using PrEP after PrEP roll-out.

Practical considerations regarding adherence are important among the users of PrEP in the phased national roll-out in Uganda. We, therefore, carried out this study to determine the level of reported adherence to PrEP and the factors associated with PrEP adherence among FSWs. We were guided by an adapted Anderson model^14^. Andersen's health belief model states that ‘people's beliefs and perceptions about benefits and barriers determines their response to the utilization of health services. Individual's access to and use of health services is considered to be a function of three characteristics; predisposing, enabling and need characteristics. Predisposing factors to utilization of PrEP included demographic characteristics such as age, educational level, marital status, religion. Enabling factors that were asked about included affordability of PrEP; social support, financial status, access to services, and distance from health facility.

Understanding these issues and identifying characteristics associated with high or low adherence is important to optimize PrEP delivery to FSWs to achieve maximum HIV prevention for this high-risk population.

## Methods

### Study design and setting

This was a cross-sectional study which was conducted at the Most at Risk Populations Initiative (MARPI) in an urban clinic which is located within the Mulago National Referral Hospital complex in Kampala. The site offers PrEP to FSWs and other key populations: MARPI is a Centers for Disease Control and Prevention (CDC) funded programme that was started in 2008 to specifically deal with health issues affecting key populations, including FSWs that are at a high risk of contracting the HIV virus^11^.

The MARPI clinic was the first public health setting to offer PrEP free of charge to FSWs in the public setting in and around Kampala. MARPI started providing PrEP in August 2017. The MARPI clinic offers friendly services to key and priority populations around Kampala including treating sexually transmitted infections, HIV prevention, care and treatment. In this paper we focus on FSWs using PrEP for HIV prevention. 15

PrEP care at the clinic is provided in accordance with Ministry of Health Uganda guidelines^16^ and Centers for Disease Control and Prevention (CDC) PrEP guidelines^17^. By December 2017, at least 3492 clients were screened for PrEP, 2123 were found eligible and 620 HIV negative clients-initiated PrEP, of whom 66% were FSWs. We defined FSWs as women who have engaged in transactional sex for money or goods within the last 60 days at the time of the interview. Participants were eligible for this study if they were ≥18 years old. This paper reports on the quantitative findings from this study on the following variables: demographics, condom use, perceived attitude of health-workers, perceived risk of HIV, drug-use, PrEP use challenges, affordability of PrEP, income level, main source of income, and number of dependents.

To study adherence to PrEP and factors affecting adherence, the sample size was calculated using (Kish Leslie, 1965) formula for cross-sectional studies.



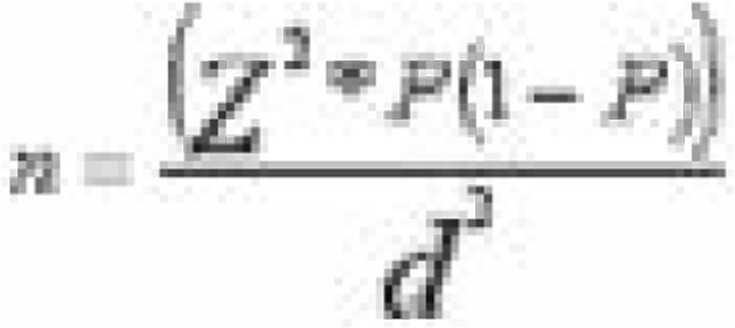



Where:

n= the desired sample size, Z = 95% confidence for point estimates of study variables; confidence interval of 1. 96, p = the proportion in the study population estimated to have characteristics being measured; among women prescribed PrEP in Uganda, adherence to PrEP by drug detection was 86%^18^.

n= (1. 962 * 0.86* 0.14)/ 0.052 =185 Participants.

In addition, to determine the sample size for this study, a review of records from MARPI clinic showed that 400 FSWs-initiated PrEP by December 2017. We used the finite population correction for proportions method to adjust the sample size (n0) based on the available population size, N=400 as per the formula below;


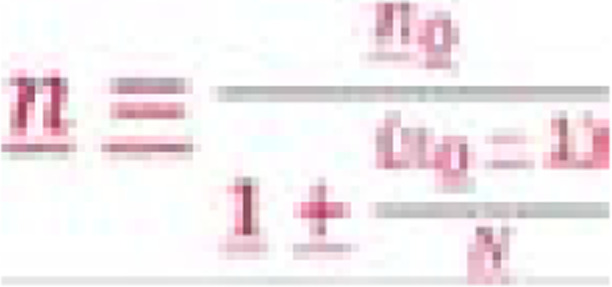



The final sample size (n) was:


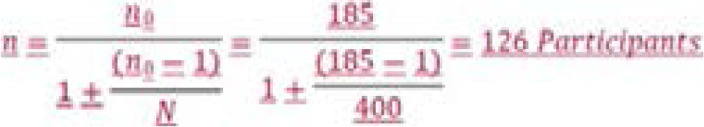



Eligible participants were FSWs aged 18 years and above that tested HIV negative according to the national testing algorithm, were eligible for and initiated PrEP and were willing to give informed consent to participate in the study.

### Exclusion criteria

Those that would otherwise be participants but were incapacitated to take part in a research study due to being very sick, mentally ill or visibly intoxicated due to alcohol use.

### Data collection methods

A systematic sampling procedure was used to recruit FSWs to interview, whereby every 2nd FSW who came to the clinic, and was eligible to take part in this study was interviewed. These women were self-identified as FSWs at the time of enrollment at the PrEP clinic, as an eligibility criterion for being given PrEP, this made it possible to identify them as sex workers. Eligibility for PrEP was documented by the clinic staff and the women were given PrEP client cards with client identification numbers written on. Before being interviewed, a FSW was asked to present her client card to verify eligibility. Furthermore, the interviewer asked the potential participans about how long they have taken PrEP for, and when they last worked as commercial sex workers to ensure that they fit the eligibility criteria.

Data was collected from 1^st^ November to 31^st^ December 2018 using interviewer administered questionnaires All participants interviewed had initiated PrEP at least two weeks before the interview. The interview with participants lasted approximately twenty minutes. A trained research assistant in Luganda, the most common local language in Kampala conducted the interviews. The researchers and research assistants received training in handling KPs especially FSWs.

We assessed adherence using a one point self-reported measure^19^. Even though self-report has been shown to overestimate pill-taking in HIV prevention trials^9^ a self-reported measure of adherence is practical in a PrEP implementation program because it is easy to collect, with minimal burden and cost for staff and the patient and the reported non-adherence is usually accurate^20^.

We asked each FSW how many doses of PrEP she had missed in the preceding seven days. Adherence was categorically defined as high adherence if a FSW took at least six doses of PrEP in a week and low adherence if a FSW took less than six doses of PrEP in a week^19^.

### Study variables

The primary outcome was adherence to PrEP among FSWs. FSWs were prescribed to take a PrEP tablet every day as per the MOH guidelines; this constituted a dose. A FSW client was expected to take seven doses of PrEP in a week. High adherence to PrEP was defined as taking at least six out of seven doses of PrEP in a week. Independent variables included age, sex, and duration of PrEP use, condom use, attitude of healthcare workers, perceived risk of HIV, drug use, contraception use, and whether one liked the color and size of PrEP pill.

### Description of variables

**Contraception:** Participants were asked if 1) they use contraception, 2) If yes, what type of contraception do they use, and 3) how long they have used that method in months.

Long-term contraception methods were grouped together for purposes of data analysis. This was a group of FSWs who use either intra-uterine devices (IUDs) or implants. This grouping was done because very few FSWs that were interviewed used long-term contraception methods, instead the majority used injectable DMPA contraception.

Condom use: Participants were asked 1) did you use a condom on your last sex encounter with a customer? 2) Did you use a condom on your last sex encounter with a non-paying sexual partner?

### Data analysis

#### Data management

The data were collected, cleaned and doubly entered into Microsoft Excel 2016. The questionnaires were labeled such that during data entry the information of a participant was verified by linking entered data, and any missing information to a participant. Continuous numerical responses were entered as absolute values while categorical responses were coded. The data was stored in a confidential manner in a password-protected computer.

Statistical analysis was performed using STATA version 15. Demographic characteristics were summarized as means and standard variations for normally distributed continuous variables, medians and 25th and 75th percentiles for skewed continuous variables and frequencies and proportions for categorical variables in Table 1. Bivariate logistic regression was first done for each independent variable. This is presented in Table 3. Variables with a p-value <0.2 were considered for multivariate analysis. To assess factors associated with high adherence to PrEP, a multivariate logistic regression was done. Variables with p-value <0.05 were considered statistically significant.

**Table 1:** Socio-demographic characteristics of the FSWs

	Frequency	%
**Age in years,** median (25^th^–75^th^ percentile)	24.5 (20.0–32.0)	
**Income in UgX**#, median (25^th^–75^th^ percentile)	100,000 (50,000–150,000)	
**Duration as sex worker (years),** median (25th–75th) percentile)	3 (1–6)	
**Number of sex partners in the last week,** (mean)	11	
**Marital status**		
Single	76	61.3
Married	13	10.5
Separated	35	28.2
**Education level** 1		
None	12	9.8
Primary	55	45.1
Secondary	50	41.0
Tertiary	5	4.1
**Religion**		
Catholics	43	34.1
Muslim	42	33.3
Protestant	41	32.5
**Age cat** đ		
18–21	42	33.3
22–30	46	36.5
31–50	38	30.2
**Contraceptive method**		
Pills	13	12.8
Long-term	14	13.7
Depo Provera	53	52.0
Condoms	22	21.6
None	24	19.1
**Sex being main income source**		
No	35	28.0
Yes	90	72.0
**Condom use with clients**		
No	20	15.9
Yes	106	84.1
**Do you have a stable sexual partner**		
Yes	78	62.0
No	48	38.0
**Condom use with non-paying stable partner**		
No	32	41.0
Yes	46	59.0
**Use of lubricant on last sexual encounter?**		
Yes	46	37.1
No	78	62.9
**Self-perceived level of risk of acquiring HIV**		
High risk	52	41.6
Moderate risk	25	20.0
Low risk	24	19.2
No risk	19	15.2
I do not know	5	4.0
**Current alcohol consumption**		
No	38	30.4
Yes	87	69.6
**Current use of drugs**		
No	80	63.5
Yes	46	36.5
**Forced to have sex in last month by anyone?**		
Yes	45	64.3
No	81	35.7
**Have you been beaten in the last month by anyone?**		
No	78	61.9
Yes	48	38.1

1 There are four missing values on education level

# 1US dollar approximates to 3500 Ug shillings

đ age categories followed the age cut-offs used at the PrEP clinic register.

**Table 3:** Bivariate and multivariate analysis of factors associated with adherence to PrEP among FSWs

Variable	Low (n=36)	High (n=90)	cOR	p-value	aOR	95% CI	p-value
Ageđ							
18–21	11 (30.6)	31 (34.4)	1				
22–30	15 (41.7)	31 (34.4)	0.73	0.51			
31–50	10 (27.8)	28 (31.1)	0.99	0.99			
Education level							
None	6 (16.7)	7 (7.9)	1				
Primary	14 (38.9)	42 (47.2)	2.57	0.138			
Secondary	16 (44.4)	40 (44.9)	2.14	0.226			
Marital status*							
Single	23 (65.7)	53 (59.6)	1		1		
Married	6 (17.1)	7 (7.9)	0.51	0.264	0.99	0.17–5.9	0.995
Separated/ Divorced	6 (17.1)	29 (32.6)	2.1	0.149	2.1	0.46–9.52	0.336
Contraception use							
No	10 (27.8)	14 (15.6)	1				
Yes	26 (72.2)	76 (84.4)	2.09	0.119			
Is sex the main source of income							
No	6 (17.1)	29 (32.2)	1				
Yes	29 (82.9)	61 (67.8)	0.44	0.097			
Used condom on last sex act with a paying customer							
Yes	24 (66.7)	82 (91.1)	1		1		
**No**	**12 (33.3)**	**8 (8.9)**	**0.2**	**0.001**	**0.07**	**0.01–0.42**	**0.003**
Used condom on last sex act with a non-paying customer							
No	14 (51.9)	18 (35.3)	1				
Yes	13 (48.2)	33 (64.7)	0.51	0.16			
Level of risk of acquiring HIV							
High risk	14 (40.0)	38 (42.2)	1				
Moderate risk	7 (20.0)	18 (20.0)	0.95	0.921			
Low risk	4 (11.4)	20 (22.2)	1.84	0.333			
No risk	8 (22.9)	11 (12.2)	0.51	0.225			
I don't know	2 (5.7)	3 (3.3)	0.55	0.539			
Counseled about PREP before initiation							
No	2 (6.1)	1 (1.1)	1				
Yes	31 (93.9)	87 (98.9)	5.61	0.165			
Do you like the color of the pill*							
Yes	23 (74.2)	80 (93.0)	1		1		
No	8 (25.8)	6 (7.0)	0.22	0.009	0.67	0.08–5.32	0.703
Do you like the packaging of the pill*							
Yes	16 (53.3)	65 (77.4)	1		1		
No	14 (46.7)	19 (22.6)	0.33	0.015	0.26	0.05–1.43	0.122
Type of contraceptive							
Pills	1 (3.9)	12 (15.8)	1		1		
**Longterm**	**6 (23.1)**	**8 (10.5)**	**0.11**	**0.061**	**0.06**	**0.004–0.77**	**0.031**
Depo Provera	11 (42.3)	42 (55.3)	0.32	0.295	0.44	0.04–5.09	0.514
Condoms	8 (30.8)	14 (18.4)	0.15	0.089	0.11	0.01–1.47	0.096

đ age categories followed the age cut-offs used at the PrEP clinic register.

* Confounder on condom use with clients “Not using condoms with clients”,

Multivariable logistic regression analysis was done to identify the factors associated with high adherence to PrEP after controlling for background variables. Assumptions for binary logistic regression, multicollinearity and outliers were assessed before fitting the multivariable logistic model. Multi-collinearity between independent variables was tested using Variance Inflation Factor (VIF) but no variable had VIF >10.

Variable was considered a confounder if it caused a change of greater than 10% to the measure of association. Variables were considered as statistically significant if the p value was less than 0.05.

## Results

### Demographic characteristics

From November to December 2018 we interviewed 126 FSWs[b9], as initially targeted from the sample size calculation. The edian age of the participants was 24.5 (IQR: 20–32) years, 41% had ever attended secondary level education (table 1). Seventy-two percent (90/125) of the participants mentioned that sex work was their main source of income[b10]. The median duration of sex work was 3 years (IQR: 1–6). Sixty one percent (76/124) of the FSWs mentioned that they were currently unmarried and had never been married.

A relatively large number of participants, 41.6 % (52/126) reported that they were at high risk of acquiring HIV, while a minority did not know their risk; 4% (5/126). The most common reason that was mentioned by 80% of the respondents about what puts them at risk of HIV was the practice of unprotected sex with a partner of unknown HIV status. Condom use at the last sexual encounter was more consistent with paying clients, 87.9% (20/126) compared to 59% (32/78) among non-paying sexual partners. The majority 96 % (118/126) of the respondents usually test for HIV many times a year, i.e. every month.

The majority of the participants, 71.4% (91/126), were categorized as having high adherence to PrEP. Most of the women did not report perfect adherence (missing 0 PrEP doses in a week) instead, only 60.98% (76/126) reported to have missed 0 PrEP doses in the past week. Some FSWs reported when they took short ‘PrEP breaks’, for example when they forgot because they went for unexpected trips, suggesting a low level of social desirability bias.

### PrEP characteristics

Majority of the respondents 97.5% (118/121) mentioned that they were counseled before initiating PrEP (table 2). Seventy-nine percent (99/121) of the respondents experienced challenges while taking PrEP, with the commonest challenge being “PrEP side-effects” which included dizziness, nausea and vomiting.

Majority of the participants 61.1% (77/126) mentioned that they spend 1 to 2 dollars (3000–5000 Uganda shillings) to access PrEP. This expenditure included money for transportation as well as buying meals on a clinic day.

Ninety-three percent (115/123) of the participants would be willing to join a PrEP adherence support group; this was described to them as a group of fellow sex-workers taking PrEP that would offer social support to each other. They could meet physically but also have a joint WhatsApp group.

Results from the bivariate analysis are presented in Table 3.

The variables that were found significant with high adherence to PrEP from the bivariate logistic regression analysis were: not using condoms with clients; [OR 0.2 (p=0.001); not liking the color of PrEP tablet; [OR 0.22(P=0.009); not liking the PrEP packaging with OR 0.33 (P=0.015) and the knowledge level about PrEP with an OR 0.42 (p=0.035).

Table 3 presents results of the final model of the multivariable logistic regression analysis.

The “use of long-term contraception” by FSWs was significantly negatively associated with high adherence to PrEP. Those who do not use condoms were less likely to adhere to PrEP.

## Discussion

We observed a relatively high adherence to PrEP at 71%. This finding means that out of 100 FSWs taking PrEP, 71 of them adhere well by taking at-least 6 out of 7 doses per week. The relatively high adherence could have been because the FSWs initiated on PrEP were at high risk for HIV infection, and hence used PrEP for protection. The level of adherence to PrEP is comparable to that in a PrEP demonstration study for sex workers in South Africa which was ranging between 70% and 85%^21^. However this adherence level to PrEP among these FSWs is slightly lower than the adherence levels seen during clinical trials of PrEP in Kenya, Uganda and South Africa where drug-level analyses revealed adherence rates of 81% – 83%7. This could have been due to high monitoring and reimbursement of those taking PrEP in clinical trials while the FSWs in this study received PrEP without any reimbursement or intense follow-up.

Our findings suggest that the relatively high level of PrEP adherence was achieved in the setting of a PrEP implementation site.. High adherence is especially important among women, as compared to men using PrEP, to achieve high efficacy of PrEP because of differences in vaginal tissue site drug levels^22^.

We found that using long-term contraception methods and not using condoms with clients were significantly negatively associated with high PrEP adherence.

Our study showed that lower adherence to PrEP has been found among FSWs who use long-term contraception methods.

Long-term contraception methods were grouped together for purposes of data analysis. This was a group of FSWs who use either intra-uterine devices (IUDs) or implants. This grouping was done because very few FSWs that were interviewed used long-term contraception methods, instead the majority used injectable DMPA contraception. This is similar to many settings in Africa where contraceptive choice is limited due to poor availability, myths about these methods, and lack of training for their administration.

Use of long-term contraception methods (implant and IUD) could have been negatively associated with PrEP adherence because of the perceived side effects of both the contraception and PrEP.

This is comparable to a clinical trial study conducted in Kenya and South Africa which found that using oral contraceptive pills at enrollment was negatively associated with good adherence to PrEP (OR: 0.37; 95% CI: 0.18 to 0.74)^23^. This clinical trial study found that adherence to PrEP was insufficient to demonstrate the effectiveness of PrEP among women whereby only 12% of participants achieved good adherence throughout their study participation. The only factor positively associated with good PrEP adherence in this study was liking the pill color. We can therefore see a link between poor adherence and contraceptive use and therefore intensified contraceptive counseling and screening before PrEP initiation and during PrEP use may improve PrEP adherence among FSWs.

This is very important as studies such as the ECHO trial are highlighting the need for integration of HIV prevention services with contraceptive services in Africa. In the ECHO trial HIV incidence was high in this population of women seeking pregnancy prevention^24^. The high uptake of contraception by FSWs in our study shows how important it is for them not to conceive while carrying out this job.

From the results, adherence to PrEP has also been found to be lower among FSWs who do not use condoms with their clients; this is alarming since PrEP is being promoted as a method of HIV prevention that should be used in combination, or as a back-up method that one can use when she fails to use condoms. In this case counselors need to emphasize the need for the FSWs to adhere to PrEP well if from their interactions they find that the use of condoms is challenging for the client. The lower adherence to PrEP among FSWs who don't use condoms with clients could have been due to poor HIV risk perception and misunderstandings about the duration of action of PrEP to protect against HIV. The health belief model^25^ explains that people will not change their health behaviors unless they believe that they are at risk; in this case, those women who do not think that they are at risk of acquiring HIV are unlikely to use a condom or take PrEP. This finding makes an interesting comparison with findings from a study among MSM using PrEP, where there was a significant increase in number of unprotected anal sex partners at the 6-month visit compared to the baseline visit^26^. In another study among MSM, risk perception was positively related with PrEP adherence, but not with condom use^27^. This furthermore underscores the need for interventions to improve the evaluation of risk perception among PrEP users. Qualitative data may better explain lower adherence among women with lower condom use.

The FSWs studied to determine the prevalence of adherence to PrEP had similar demographic characteristics (age, education,) to the FSWs in Kampala, Uganda that were studied to determine the burden and characteristics of HIV infection in this population^5^.

Similar to the iPrEX study, a high proportion of participants in our study mentioned that they experienced side-effects mainly gastrointestinal complaints such as nausea and ulcer pain^28^.

A qualitative study done among women in the US before PrEP implementation showed that the women were mostly willing to use PrEP, however key barriers to PrEP uptake included distrust of the medical system, stigma, and cost. Findings suggested that US women viewed PrEP as an important prevention option, assuming side effects and the cost to the consumer are minimal, the efficacy of the drug is reasonable, and PrEP is delivered by trusted providers in trusted venues^29^. In this study the investigators looked at a population of women in a developing country, after PrEP rollout. PrEP was offered at no cost to the FSWs who took part in this study, however they incurred costs to go to the clinic or reach the outreach facility where PrEP was being distributed. A high number of the women mentioned that they experienced side-effects of PrEP especially in the first month of initiating PrEP, they mentioned that this affected their adherence negatively however it was not statistically significant. This might be because the women who completely stopped taking PrEP because of unbearable side-effects were no longer coming for PrEP refills and the ones interviewed were therefore the resilient ones. This idea was explored deeper in the qualitative interviews.

In a study that explored the potential benefits of integrating PrEP into combination HIV prevention for FSWs and the likely challenges to implementation, it was noted that FSWs face particular structural challenges to PrEP use, including stigmatizing health services; fear of disclosure to other FSWs and clients; fear of the authorities and a lack of social support^30^. In this study the FSWs mentioned that they would like to have adherence support from their peers who are also using PrEP. This is a way to overcome the challenge of lack of social support.

## Strength and limitations

Our findings on PrEP adherence in FSWs may be applicable to FSWs in other settings, particularly in the developing world and in other groups with high rates of transactional sex.

The duration of PrEP use among the participants varied significantly and thus reflects adherence outcomes for longer-term use of PrEP as well. We highlight a limitation of this study in regards to objectivity of the adherence measure. Objective measures such as pill counts, drug levels in hair, urine or blood samples, and electronic device monitoring would better report adherence however this study used a measure of adherence which is easily replicable in the resource limited real world setting. This study also includes a small number of FSWs recruited from one facility through systematic random sampling; this could mean that the study participants are less representative of the underlying population of women.

## Conclusion and implications

The findings from this study show a high proportion of FSWs has high self-reported adherence to PrEP in this setting. However, barriers to PrEP adherence such as use of long-term contraception need to be addressed for successful PrEP implementation to improve adherence going forward. There is need for service care providers to reinforce positive behaviors such as use of other HIV prevention measures such as condoms devotedly when there is a break from taking PrEP.

Service care providers need to reinforce positive behaviors such as use of condoms devotedly during a ‘PrEP break’ when they are not taking PrEP to avoid sero-conversion. Intensified contraceptive counseling and screening may improve PrEP adherence among FSWs.

## Figures and Tables

**Table 2:** PrEP adherence characteristics of FSWs

Variable	Frequency	%age
**Counselled before PrEP Initiation** 2		
No	3	2.5
Yes	118	97.5
**Do you encounter challenges while taking PrEP?**		
No	26	20.8
Yes	99	79.2
**Challenges encountered with PrEP**		
Pill burden	6	6.1
Stigma	7	7.1
Have to hide while taking pills	5	5.1
Forget to take pills	5	5.1
Size of the pills	3	3.0
Side effects	73	73.7
**Duration on PrEP** 3		
1–3 months	50	43.5
4–6 months	32	27.8
>6 months	33	28.7
**Like pill color** 4		
No	14	12.0
Yes	103	88.0
**Like pill size** 5		
No	43	37.1
Yes	73	62.9
**Like Pill packaging** 6		
No	33	29.0
Yes	81	71.0
**Expenditure to access PrEP**		
3000–5000	77	61.1
5500–8000	14	11.1
>8500	35	27.8
**Can you afford PrEP if it's not free?**		
No	59	47.2
Yes	66	52.8
**Perceived attitude of health workers**		
Poor	3	2.5
Fair	5	4.1
Good	49	40.2
Very good	65	53.3
**Would you like to join a PrEP adherence group?**		
No	8	6.5
Yes	115	93.5
**Would you like to receive message reminders about PrEP**		
No	13	10.7
Yes	108	89.3

2 There are 3 missing values on counseled before PrEP initiation

3 There are 11 missing values on duration on PrEP

4 9 missing values on “Pill color”

5 10 missing values on “Pill size”

6 12 missing values on “Pill packaging”

## List of abbreviations

IRB: Institutional Review Board
HDREC: Higher Degrees Research and Ethics Committee
HIV: Human Immuno-deficiency Virus
MSM: Men who have sex with men
FSWs: Female Sex workers
REC: Research Ethics Committee
MARPs: Most at Risk Persons
TDF/FTC: Tenofovir disoproxil fumarate/emtricitabine
PEP: Post Exposure Prophylaxis
PrEP: Pre-Exposure Prophylaxis
WHO: World Health Organization

## Declarations

### Availability of data and material

The datasets used and/or analyzed during the study are available from the corresponding author upon reasonable request.
